# Recent Advances in Radiopharmaceuticals for Cancer Diagnosis and Therapy

**DOI:** 10.32604/or.2026.075923

**Published:** 2026-03-23

**Authors:** Ye Ri Han, Sang Bong Lee

**Affiliations:** 1 Department of Chemistry, Duksung Women’s University, Seoul, Republic of Korea; 2 SimVista Inc., A-13, 194-25 Osongsaengmueong1-ro Osong-eup Heungdeok-gu Chungcheongbuk-do, Cheongju-Si, Republic of Korea; 3 Department of Biomedical Sciences, Chonnam National University Medical School, 264, Hwasun-eup, Hwasun-gun, Jeollanam-do, Republic of Korea

**Keywords:** Radiotheranostics, radioligand therapy, alpha emitters, terbium-161, prostate-specific membrane antigen, somatostatin receptor, patient stratification, dosimetry, precision oncology

## Abstract

Radiopharmaceuticals deliver diagnostic or therapeutic radionuclides to disease sites with molecular precision. Over the past five years, clinical adoption has accelerated, led by U.S. Food and Drug Administration approvals of ^177^Lu-DOTA-TATE and ^177^Lu-PSMA-617 and their complementary Positron Emission Tomography agents (^68^Ga-DOTA-TATE, ^68^Ga-PSMA-11), which have established radiotheranostics as a pillar of oncology care. The new generation of agents couples optimized radionuclides (β^−^, α, and Auger emitters) to antibodies, peptides, and small-molecule vectors that improve tumor uptake, residence time, and clearance profiles, thereby enhancing efficacy and safety. Beyond neuroendocrine tumors and prostate cancer, radiotheranostic strategies are advancing for diverse malignancies by exploiting tumor-specific antigens, overexpressed receptors, and intracellular targets. Notably, α-emitters such as ^225^Ac and ^211^At—owing to high linear energy transfer and short path length—show potent cytotoxicity with limited off-target injury, while emerging β/Auger emitters like ^161^Tb may surpass ^177^Lu in microdosimetric effectiveness. Concurrent innovations in patient selection and response prediction leverage diagnostic radiopharmaceuticals for image-guided stratification, individualized dosimetry, and adaptive treatment planning, supporting the broader paradigm of precision medicine. Although oncology remains the primary focus, applications are expanding to neurodegeneration, cardiovascular disease, and inflammatory conditions. This review synthesizes technological and clinical progress from 2021–2025, spanning FDA-approved and late-stage investigational agents; mechanisms of radiopharmaceutical-induced cell death; dosimetry methodologies; trial landscapes for expanding indications; and translational challenges, including supply chains, chelation chemistry, and toxicity management. Accordingly, this review focuses on the latest radiopharmaceutical diagnostic and therapeutic technologies, integrating advances in radionuclide platforms, targeting vectors, dosimetry, and clinical trial data from 2021–2025 to guide future development and clinical implementation of precision radiotheranostics.

## 1 Introduction

**Figure 1:**
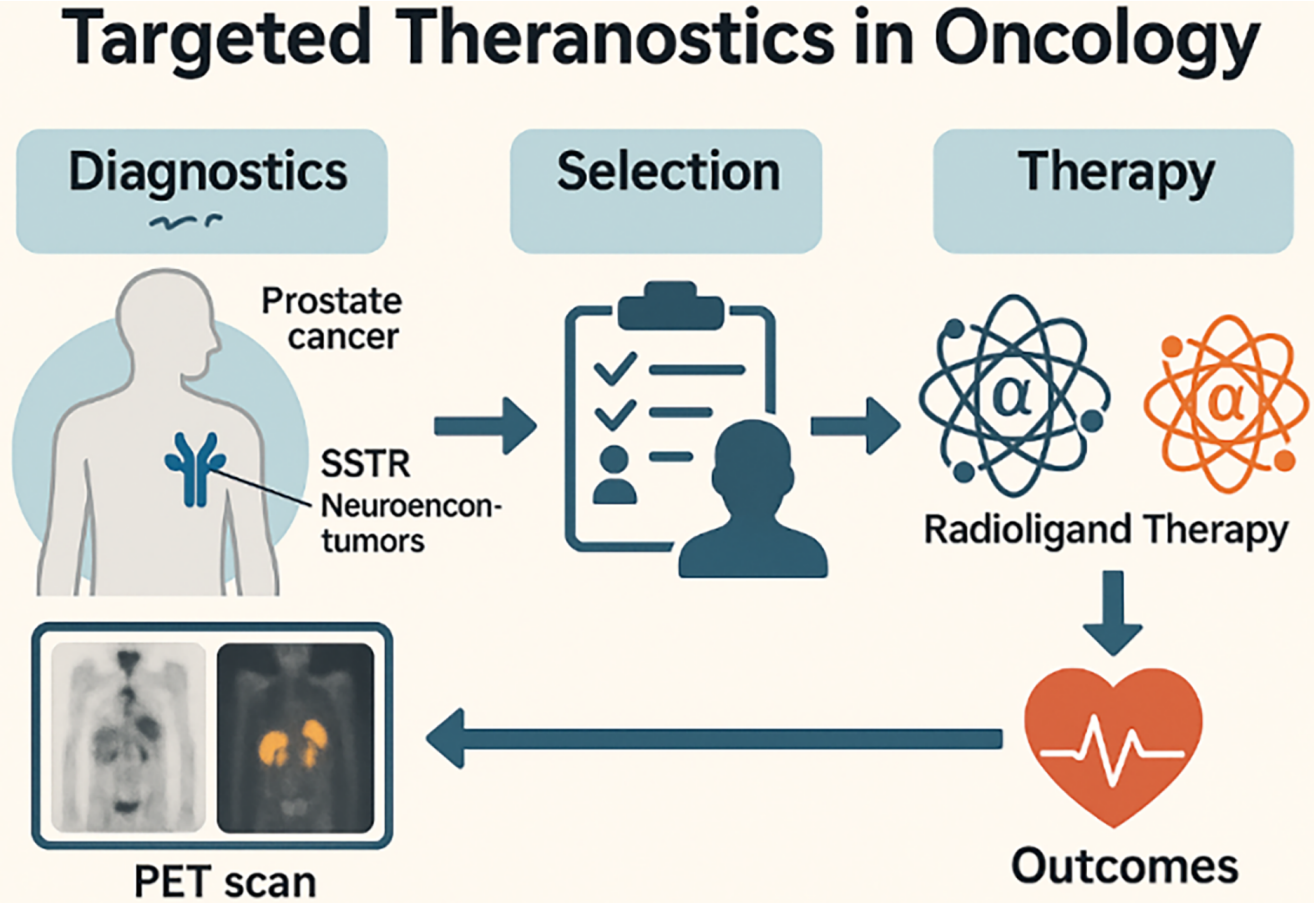
Targeted theranostics workflow in oncology. Schematic depicting the theranostic pathway from Diagnostics to Outcomes. Left, Diagnostics: PET imaging identifies target expression in tumors—illustrated for PSMA (prostate cancer) and SSTR (neuroendocrine tumors). Middle, Selection: patient eligibility is assessed based on imaging positivity and clinical criteria. Right, Therapy: eligible patients receive radioligand therapy (β− or α emitters, not specified here). Bottom, Outcomes: treatment response and safety are monitored; results feed back to re-image/re-assess, closing the theranostic loop. Schematic created using corel draw. Note: PSMA, prostate-specific membrane antigen; SSTR, somatostatin receptor; PET, positron emission tomography

Radiotheranostics, the coordinated use of matched diagnostic and therapeutic radiopharmaceuticals directed to the same molecular target—has emerged as a clinically actionable framework for precision oncology. By linking noninvasive target confirmation with individualized activity planning and on-treatment response assessment, radiotheranostics enables a closed-loop workflow that spans patient selection, image-based dosimetry, and longitudinal outcome evaluation [1–3]. Its clinical feasibility has been validated most notably along the somatostatin receptor (SSTR) and prostate-specific membrane antigen (PSMA) axes, where positron emission tomography (PET) tracers (e.g., ^68^Ga- or ^18^F-labeled ligands) are paired with therapeutic agents such as ^177^Lu, establishing components of standard care in selected indications [4–6] (Fig. 1).

Radiopharmaceutical vectors—including small molecules, peptides, and antibodies—deliver radionuclides to disease sites with high specificity. When coupled to PET or single-photon emission computed tomography (SPECT), they afford sensitive whole-body lesion mapping; when labeled with therapeutic emitters, they deposit cytotoxic energy locally, producing lethal DNA damage within targeted cells while limiting dose to noninvolved tissues. Relative to external-beam radiotherapy, which necessarily traverses healthy structures, radioligand therapy (RLT) confines dose to molecularly defined compartments, allowing therapeutic indices to be improved with comparatively low mass of vector [7–9]. Although development has been led by oncologic applications, expansion into neurodegenerative, cardiovascular, and inflammatory disorders is ongoing as disease-defining targets and quantitative imaging methods mature [10,11].

The physical and radiobiological characteristics of the emission determine clinical design. Beta (β) emitters such as ^177^Lu exhibit low linear energy transfer (LET; ~0.2 keV/μm) and millimeter-scale ranges that confer a beneficial cross-fire effect in heterogeneous tumors. Alpha (α) emitters (e.g., ^225^Ac, ^211^At, ^212^Pb, ^212^Bi) deliver very high LET (≈50–230 keV/μm) over tens of micrometers, producing dense ionization tracks well suited to micrometastatic or small-volume disease and to hypoxic niches that are relatively resistant to low-LET radiation. Auger electron emitters offer subcellular-scale lethality but require nuclear-proximal localization for maximal effect, which has constrained clinical adoption to date [12–15].

Two domains currently drive the field’s acceleration. First, isotope innovation is advancing toward translation. Terbium-161 (^161^Tb), which co-emits abundant low-energy electrons with β-particles, has shown potential efficacy advantages over ^177^Lu in select targets, including SSTR and PSMA. In α-therapy, labeling with ^225^Ac, ^212^Pb, or ^211^At has yielded encouraging preclinical and early clinical signals; exemplar programs include sodium astatide (^211^At-NaAt) for iodine-refractory thyroid cancer and emerging ^211^At-PSMA constructs [16–19]. Second, target and imaging diversification is expanding the addressable population. Beyond ^18^F-FDG, advances in PET hardware (e.g., silicon-photomultiplier–based systems, total-body PET) and kinetic/dynamic modeling enhance sensitivity and quantification, while a growing repertoire of tumor-associated targets—such as fibroblast activation protein (FAP), gastrin-releasing peptide receptor (GRPR), TROP-2, Nectin-4, L-type amino acid transporter 1 (LAT1), glypican-1 (GPC-1), and EphA2—are entering preclinical and early clinical evaluation, poised to complement or extend PSMA/SSTR frameworks [20–23].

Despite momentum, several translational determinants will govern breadth of adoption. Robust, standardized, patient-specific dosimetry is needed to optimize activity selection and scheduling across isotopes and targets, motivating protocols that leverage the same ligand labeled with diagnostic and therapeutic nuclides for individualized planning. In parallel, reliable supply chains for α-emitters (e.g., ^225^Ac, ^211^At), continued improvements in quantitative imaging and reconstruction, and integrated, multidisciplinary care pathways will be essential to ensure safety, scalability, and equitable access [24–26].

**Figure 2:**
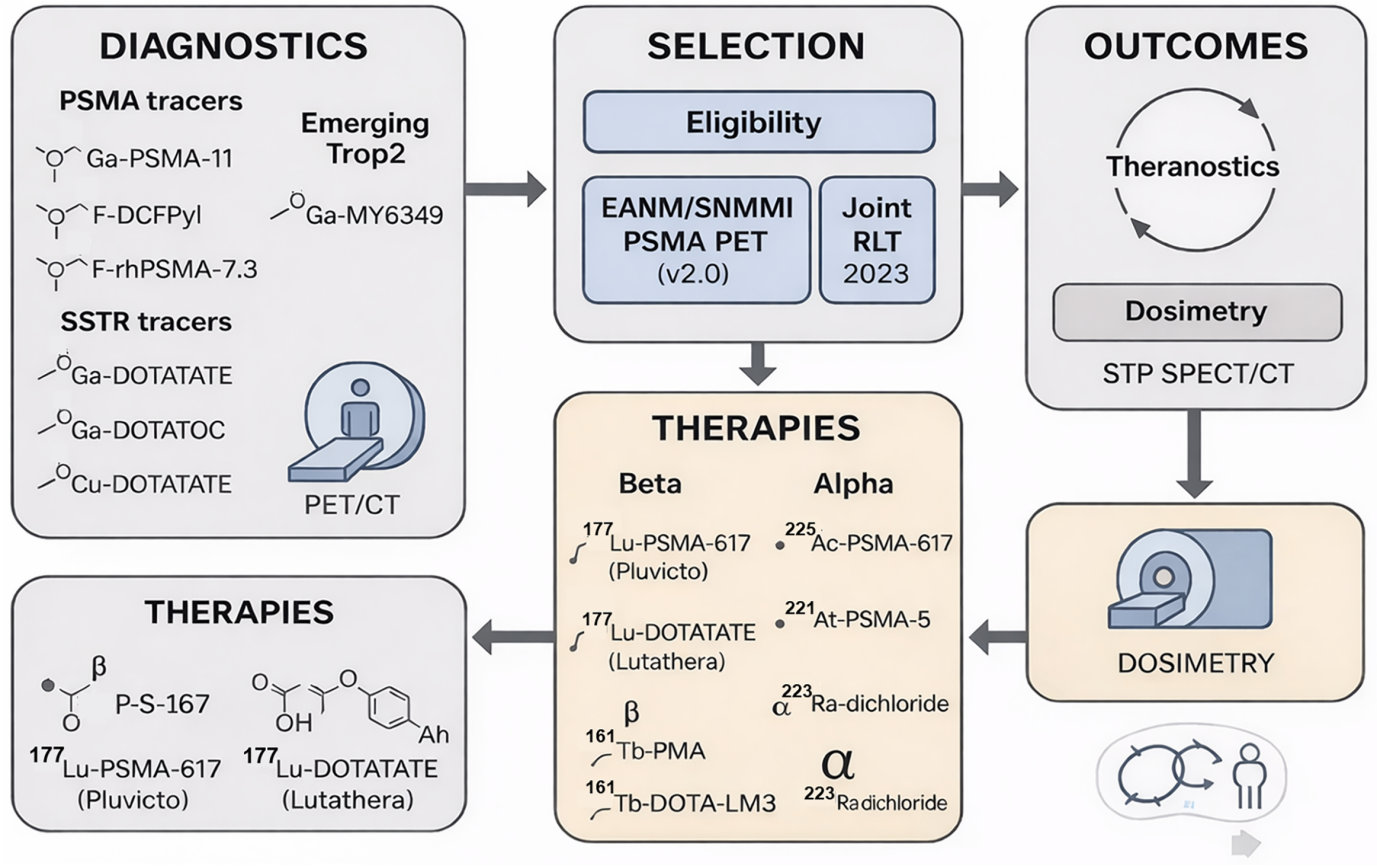
Theranostics in Oncology—PSMA & SSTR pathways with emerging Trop2. Diagnostics–Selection–Therapy overview. Left panel lists PSMA PET tracers (^68^Ga-PSMA-11, ^18^F-DCFPyL, ^18^F-rhPSMA-7.3, ^18^F-PSMA-1007) and SSTR PET tracers (^68^Ga-DOTATATE, ^68^Ga-DOTATOC). An emerging target (Trop2) is illustrated with ^68^Ga-MY6349. The central panel depicts patient selection guided by major practice documents (e.g., EANM/SNMMI PSMA PET v2.0 and NANETS/SNMMI PRRT 2020). The right panel summarizes radioligand therapy (RLT) options grouped by emission type: β^−^ therapies (^177^Lu-PSMA-617 [Pluvicto], ^177^Lu-DOTATATE [Lutathera], ^161^Tb-PSMA, ^161^Tb-DOTA-LM3) and α therapies (^225^Ac-PSMA-617, ^211^At-PSMA-5, ^223^Ra-dichloride). Outcome icons indicate OS/PFS tracking and patient-specific dosimetry. Procedure flow and dosimetry loop. A systems flowchart reiterates the diagnostic imaging → eligibility assessment → therapy (β/α) → outcomes cycle, emphasizing the theranostic loop and integration of single-time-point (STP) SPECT/CT dosimetry to inform subsequent treatment decisions. Schematic created using corel draw and chem draw. Note: PSMA, prostate-specific membrane antigen; SSTR, somatostatin receptor; PET/CT, positron emission tomography/computed tomography; RLT, radioligand therapy; PRRT, peptide receptor radionuclide therapy; OS, overall survival; PFS, progression-free survival; EANM, European Association of Nuclear Medicine; SNMMI, Society of Nuclear Medicine and Molecular Imaging; NANETS, North American Neuroendocrine Tumor Society

In summary, building upon the clinical success of SSTR- and PSMA-directed platforms, radiotheranostics is evolving toward a pan-tumor precision-medicine paradigm enabled by high-LET α-emitters and mixed-emission nuclides, diversified target portfolios, and increasingly rigorous image-guided dosimetry. Against this backdrop, the present review aims to provide an integrated, clinically oriented overview of contemporary radiotheranostic practice and emerging technologies, spanning radionuclide platforms (β^−^, α, and Auger emitters), targeting vectors and antigens, and quantitative PET/SPECT methodologies for individualized dosimetry and response assessment. Particular emphasis is placed on expanding targets and disease spaces, including not only SSTR- and PSMA-based agents but also fibroblast activation protein (FAP/FAPI) radiotheranostics, gastrin-releasing peptide receptor (GRPR) ligands, HER2-directed imaging and therapy, and other next-generation targets such as TROP-2, Nectin-4, LAT1, GPC-1, and EphA2. We further highlight practical considerations and pitfalls—encompassing patient selection, activity planning, supply-chain and manufacturing constraints, and toxicity management—and delineate opportunities by which next-generation radiopharmaceuticals and matched diagnostic–therapeutic pairs may reshape therapeutic standards within and beyond oncology [10,27] (Fig. 2).

## 2 Physical and Radiobiological Principles of Therapeutic Emissions

Beta (β), alpha (α), and Auger electron emitters differ fundamentally in linear energy transfer (LET), track length, and dominant modes of DNA injury—differences that directly condition patient selection, treatment planning, and expected toxicities. α-emitters typically release 4–8 MeV particles with high LET (≈50–230 keV/μm) over tens of micrometers, creating dense ionization tracks that favor eradication of micro metastases and hypoxic tumor niches with limited collateral injury when properly targeted. By contrast, β-emitters have low LET (≈0.1–10 keV/μm) and millimeter-scale ranges, enabling crossfire within heterogeneous lesions but relying more on indirect, Reactive oxygen stress-mediated DNA damage. Auger emitters deliver extremely short-range, high-LET electrons at subcellular scales; their therapeutic index depends on achieving nuclear-proximal localization to exploit clustered double-strand breaks. These physical–biologic distinctions, summarized across approved and investigational isotopes, underpin the modern palette of radiopharmaceutical design [12,26].

**Figure 3:**
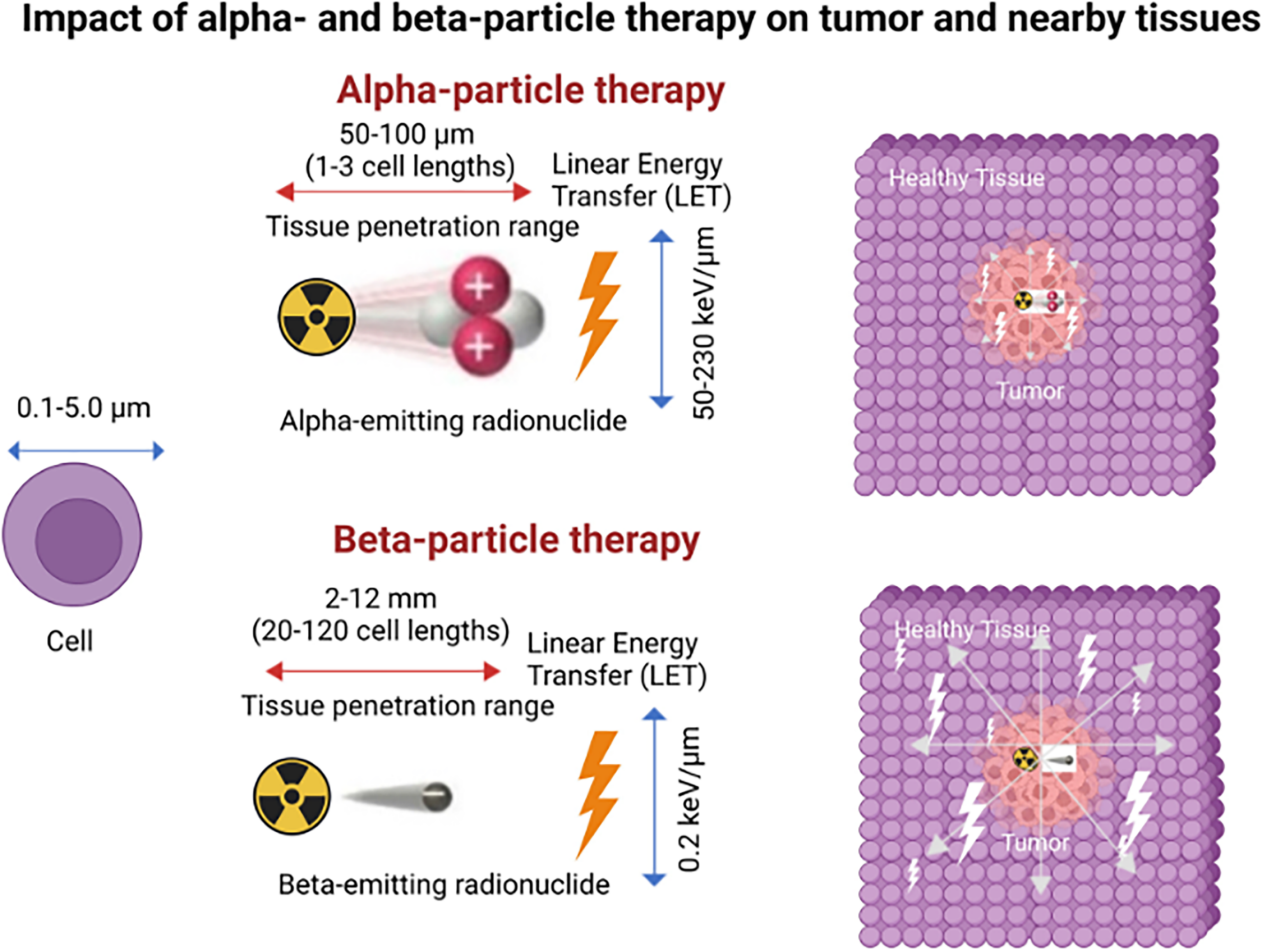
Targeted α-particle and β-particle therapy comparison. Illustration shows the characteristic features of α and β particles. α Particles are positively charged particles composed of two protons and two neutrons, essentially the nucleus of a helium atom, and β particles are negatively charged particles, essentially electrons. α Particles have much greater mass, higher linear energy transfer (LET), travel a much shorter distance in tissue, and are more cytotoxic than β particles. The illustration includes specific values of these characteristics for reference but is not to scale. The schematic and figure have been adapted with permission from previously published figures and concepts in reference [12]; copyright 2023. Reproduced from Burkett BJ et.al., Radiology: Imaging Cancer, under CC By 4.0. © RSNA, 2023 (Open access; journal policy indicates CC BY-NC-ND 4.0.)

In clinical workflows, these emission properties are operationalized through theranostic pairing: an imaging agent confirms target expression and biodistribution, then its therapeutic counterpart delivers the cytotoxic payload to the same epitope. This closed-loop approach supports patient triage, activity planning, and on-treatment response assessment within a single molecular framework [12] (Fig. 3).

## 3 Diagnostic Innovations in Oncology PET

Hardware advances—most notably silicon–photomultiplier detectors, extended axial field-of-view/“total-body” PET, time-of-flight improvements, and routine quantitative calibration pipelines—are expanding sensitivity and enabling consistent longitudinal quantification. In parallel, standardized reconstruction, harmonized SUVs, and growing use of kinetic/dynamic analyses (e.g., parametric Ki maps) are improving response assessment beyond simple lesion detectability. Together with a widening portfolio of non-FDG tracers, these developments are accelerating precision phenotyping and treatment monitoring across tumor types [23].

TROP-2 imaging. As a prototypical pan-tumor program, TROP-2 PET has advanced rapidly from preclinical validation to first-in-human studies. The small-molecule tracer [^68^Ga]MY6349 demonstrated high and heterogeneous uptake across multiple histologies, consistent with broad TROP-2 expression, and enabled early detection of antibody–drug conjugate (ADC) response in triple-negative breast cancer—supporting its use as a companion diagnostic to guide patient selection, sequence therapies, and monitor pharmacodynamic effects during the first treatment cycles [23,24]. Platform-level learnings include: (i) the feasibility of whole-body target mapping to capture inter- and intra-patient heterogeneity; (ii) the potential of semi-quantitative thresholds (e.g., SUV_{peak}/tumor-to-background ratio) to enrich for ADC responders; and (iii) opportunities to integrate temporal readouts (baseline → early on-treatment PET) as predictive biomarkers. Preclinical ^89^Zr/^177^Lu-anti-TROP-2 constructs further underline theranostic potential, suggesting that image-positive disease could be candidates for radiolabeled TROP-2 therapy where dosimetry and off-target risks are acceptable [23,24].

Nectin-4 PET. Nectin-4–directed imaging has likewise gained traction. First-in-human studies with [^68^Ga] N188 in advanced urothelial carcinoma showed lesion uptake correlating with immunohistochemical expression, supporting the biological specificity of the signal; exploratory cohorts across additional epithelial cancers suggest broader applicability that could seed Nectin-4 theranostic development [23]. Clinically, this axis is attractive because (i) Nectin-4 is already a drugged target (e.g., ADCs), enabling cross-modal triangulation of target engagement; (ii) PET can map heterogeneity at presentation and on treatment (e.g., emergent Nectin-4–low clones); and (iii) imaging may inform sequencing between ADCs and prospective radioligand approaches. Key developmental questions include optimal imaging time-points, thresholds for positivity linked to outcomes, and mitigation of physiologic/background uptake that may affect detection in heavily pretreated patients [23].

Expanding pan-tumor targets. Beyond TROP-2 and Nectin-4, multiple programs are moving through optimization and early clinical testing. LAT1 (amino-acid transport), GPC-1 (cell-surface heparan sulfate proteoglycan), EphA2 (receptor tyrosine kinase), and carbonic anhydrase IX (CA IX, hypoxia-linked carbonic anhydrase) exemplify agnostic biomarkers with cross-histology prevalence and plausible radiotheranostic translation if biodistribution and target-to-organ ratios prove favorable [24]. For each, the translational logic is similar: (i) demonstrate robust PET signal with correlation to tissue expression and/or pathway activity; (ii) characterize normal-organ kinetics (renal/hepatic/salivary/intestinal) to bound safety margins for potential therapy; and (iii) establish quantitative cut-points and repeatability (test–retest, EARL-style harmonization) to qualify imaging as an enrichment or response biomarker in multicenter trials [23].

Practical considerations and pitfalls. Across these programs, three themes recur:

1. Biology in motion. Target abundance can be therapy-induced (up- or down-regulated) and spatially heterogeneous; serial PET provides a noninvasive window to adapt therapy but requires harmonized acquisition to separate biology from noise [23].
2. Quantitative endpoints. While SUV_{max}/SUV_{peak} remain pragmatic, lesion-level metrics (metabolic/volumetric burden) and dynamic/parametric readouts may better capture early pharmacodynamic effects and predict outcome, particularly for ADC-like mechanisms [23,24]. However, SUV_max and SUV_peak are semi-quantitative measures that are sensitive to acquisition and reconstruction protocols, lesion size, and image noise, and these sources of variability propagate into non-trivial uncertainty when such metrics are used as surrogates for absorbed dose or early response. By contrast, full kinetic or parametric modeling—although more resource-intensive—can better disentangle delivery, binding, and retention, and may therefore provide more robust inputs for dosimetry calculations and pharmacodynamic response assessment [28–31].
3. Theranostic readiness. Image-positive disease does not automatically equal therapeutic index; translation demands dosimetry feasibility, manufacturability (chelation/linker stability), and organ-at-risk constraints compatible with repeated dosing [23].

**Figure 4:**
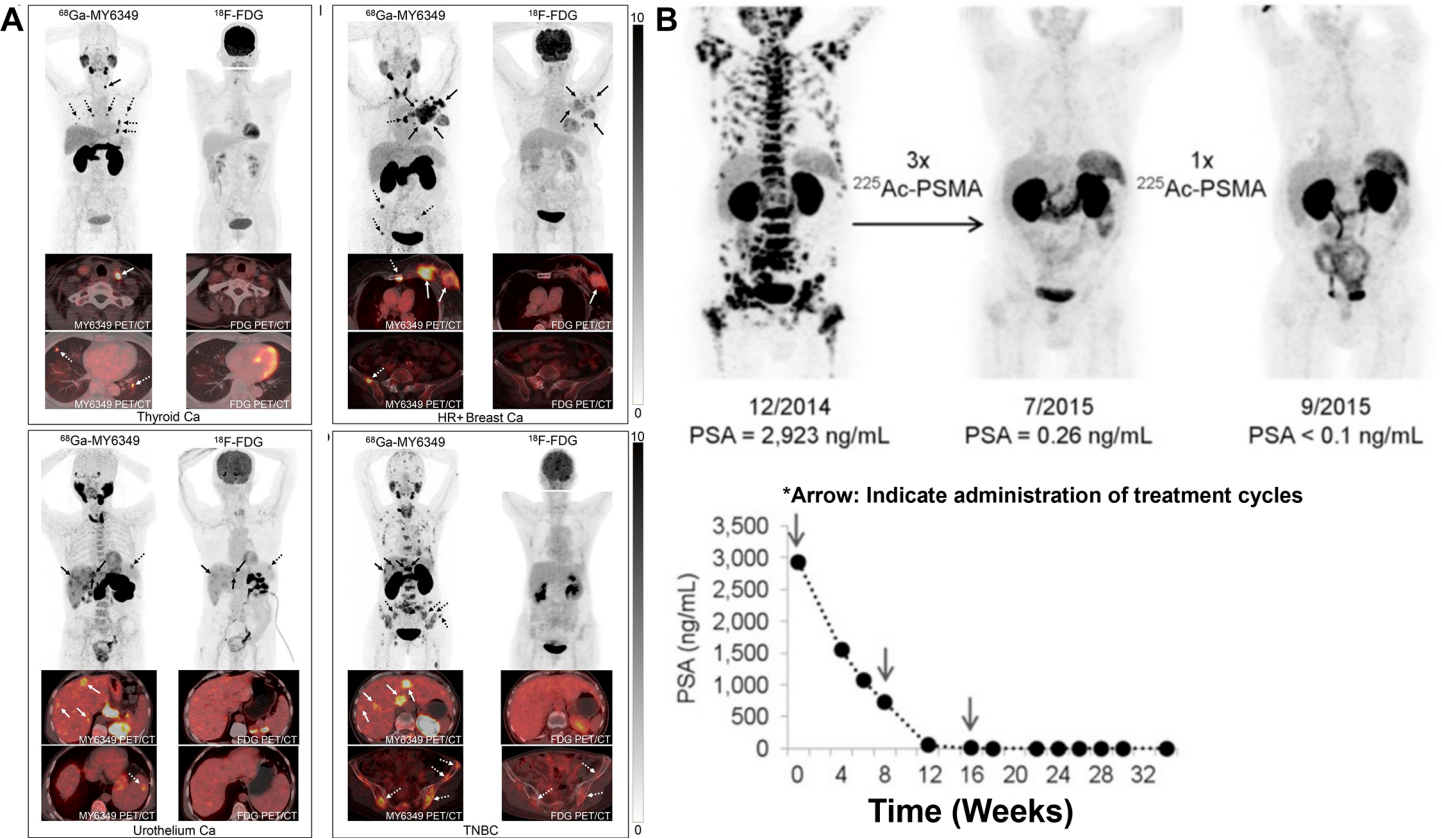
Clinical PET/CT illustrations of Tumor Imaging and PSMA-Directed Theranostic Response. (**A**) Representative ^18^F-FDG PET/CT and ^68^Ga-MY6349 PET/CT images in patients with various cancers [24], *Arrow: Indicate diseased region. (**B**) [^68^Ga]PSMA-11 PET/CT scans in a metastatic castration-resistant prostate cancer patient with diffuse bone metastases after administration of [^225^Ac]PSMA-617, *Arrow: Indicate administration of treatment cycles. Complete remission was achieved after four cycles of treatment (Cited from reference No. 79 in accordance with the open access policy [23]. The schematic and figure have been adapted with permission from previously published figures and concepts in references [23,24]; copyright 2025, respective copyright holders. Reproduced from Watabe T, Hirata K, Iima M, et al. Recent advances in theranostics and oncology PET: emerging radionuclides and targets. Ann Nucl Med. 2025;39(9):909–921. 10.1007/s12149-025-02090-z. Licensed under the Creative Commons Attribution 4.0 International License (CC BY 4.0). Adapted from Chen H, Zhao L, Pang Y, et al. ^68^Ga-MY6349 PET/CT imaging to assess Trop2 expression in multiple types of cancer. J Clin Invest. 2025;135(1):e185408. 10.1172/JCI185408. © 2024 Chen et al. Licensed under CC BY 4.0; changes were made

Collectively, these innovations position non-FDG PET not only as a staging tool but as a decision platform—triaging patients to targeted drugs and, where appropriate, enabling a direct diagnostic-to-therapeutic transition along the same molecular axis [23,24] (Fig. 4). Overall, Fig. 4 highlights how PET/CT radiotracers enable visualization of tumor biology across cancer types and can also be used to monitor and demonstrate treatment response following targeted radioligand therapy.

## 4 Therapeutic Radionuclides: Recent Advances

### 4.1 **β** and Mixed-Electron Emitters

β and mixed-electron emitters. Among β-emitters, ^177^Lu remains the clinical workhorse for peptide and small-molecule ligands because its 6.65-day half-life, favorable β^−^ spectrum, and clean DOTA-based radiochemistry align well with centralized production and multi-day logistics. By comparison, terbium-161 (^161^Tb) offers a Lu-177–like half-life (~6.9 days) and comparable labeling conditions with DOTA chelators but differs in its decay scheme: in addition to β^−^ particles, ^161^Tb co-emits abundant low-energy conversion/Auger electrons. Microdocumentary modeling and head-to-head preclinical work suggest that these short-range electrons increase sub-millimeter energy deposition—potentially improving tumor-to-normal dose at the cellular and micro metastatic scale, particularly when target expression is heterogeneous or lesion size is small [12,15–17,23]. Practically, this means many ^177^Lu ligands can be “isotope-switched” to ^161^Tb with minimal synthetic re-engineering (same DOTA scaffold, similar labeling temperatures), while gaining additional short-range dose from the electron component [12,15–17].

From an imaging/quantification standpoint, ^161^Tb emits low-energy photons (e.g., ~49–75 keV) that enable SPECT readout; although scatter and collimation choices differ vs. ^177^Lu, first patient studies have shown technically feasible lesion visualization and post-therapy dosimetric sampling [17,23]. Early clinical signals now span two major theranostic axes: (i) PSMA, where first-in-human ^161^Tb-PSMA SPECT/CT confirmed tumor targeting with acceptable acute tolerability, and (ii) SSTR, where ^161^Tb-labeled SSTR antagonists (e.g., DOTA-LM3) have demonstrated initial safety/feasibility in patients and superior preclinical efficacy vs. ^177^Lu analogs, consistent with the enhanced micro dosimetry hypothesis [16–18,23]. Importantly, the organ-at-risk profile appears broadly similar to ^177^Lu for kidneys and marrow in early experience, while any incremental salivary/renal risk from the electron component is being evaluated in ongoing dosimetry-rich protocols [16–18,23,27,31].

**Figure 5:**
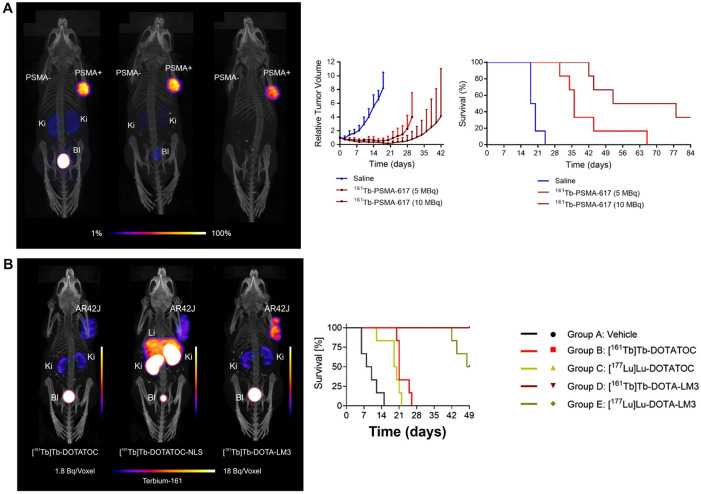

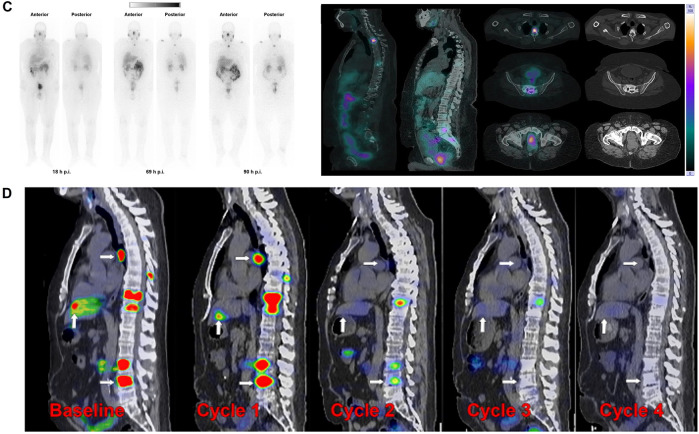
Translational SPECT/CT Theranostics with ^161^Tb and ^177^Lu-PSMA in Prostate Cancer. (**A**) SPECT/CT images of mice after of Terbium-161 for PSMA-targeted radionuclide therapy of prostate cancer [31], (**B**) Combination of terbium-161 with somatostatin receptor antagonists—a potential paradigm shift for the treatment of neuroendocrine neoplasms [12]. (**C**) Whole-body images at different time points after injection. Representative SPECT/CT sagittal and axial slices and CT axial slices demonstrating physiological biodistribution of ^161^Tb-PSMA in lacrimal, parotid, and submandibular glands; nasopharyngeal mucosa; liver; intestinal tract; kidneys; and urinary bladder, as well as pathologic uptake in primary prostate tumor and metastatic bone lesions. p.i. = after injection [17]. (**D**) Posttherapy monitoring of index lesions with SPECT/CT imaging. Fused SPECT/CT sagittal images in a 56-year-old man with prostate-specific membrane antigen (PSMA)–avid metastatic prostate cancer undergoing lutetium 177 (^177^Lu) PSMA-617 therapy [27]. The schematic and figure have been adapted with permission from previously published figures and concepts in references [12,17,27,31]; copyright 2022–2024, respective copyright holders. Adapted from Begum NJ, Glatting G, Wester HJ, Eiber M, Beer AJ, Kletting P. The effect of ligand amount, affinity and internalization on PSMA-targeted imaging and therapy: a simulation study using a PBPK model. Scientific Reports. 2019;9:20041. 10.1038/s41598-019-56603-8. Licensed under CC BY 4.0 (https://creativecommons.org/licenses/by/4.0/). Reproduced from Burkett BJ, Bartlett DJ, McGarrah PW, et al. A review of theranostics: perspectives on emerging approaches and clinical advancements. Radiology: Imaging Cancer. 2023;5(4):e220157. 10.1148/rycan.220157. © RSNA, 2023 (Open access; journal policy indicates CC BY-NC-ND 4.0.). Licensed under CC BY-NC-ND 4.0 (https://creativecommons.org/licenses/by-nc-nd/4.0/). Reproduced with permission from Al-Ibraheem A, Doudeen RM, Juaidi D, Abufara A, Maus S. ^161^Tb-PSMA radioligand therapy: first-in-humans SPECT/CT imaging. Journal of Nuclear Medicine. 2023;64(8):1322–1323. 10.2967/jnumed.122.265291). Reproduced from Busslinger SD, Mapanao AK, Kegler K, et al. Comparison of the tolerability of ^161^Tb- and ^177^Lu-labeled somatostatin analogues in the preclinical setting. European Journal of Nuclear Medicine and Molecular Imaging. 2024;51:4049–4061. 10.1007/s00259-024-06827-2. Licensed under CC BY 4.0 (https://creativecommons.org/licenses/by/4.0/)

On the manufacturing side, ^161^Tb production has matured via reactor-based routes with post-irradiation chemical separation and standardization of quality attributes (radionuclidic purity, specific activity) suitable for clinical translation. This, coupled with the plug-and-play DOTA chemistry, supports scale-up to multi-center studies using existing ^177^Lu supply chains as a template [15–17,23,27,31]. Taken together, these attributes position ^161^Tb as a compelling alternative to ^177^Lu—particularly for small-volume lesions or heterogeneous targets—while preserving the operational advantages that enabled ^177^Lu’s widespread adoption [15–18,23,27,31] (Fig. 5). Overall, Fig. 5 illustrates the theranostic utility of SPECT/CT using ^161^Tb-based agents by demonstrating treatment application, normal-organ biodistribution, and longitudinal lesion monitoring to support therapy planning and response assessment.

### 4.2 Targeted Alpha Therapy

Clinical momentum in targeted α-therapy now spans ^225^Ac, ^212^Pb (via *in vivo* decay to ^212^Bi), and ^211^At, with early clinical signals observed across several tumor types. In neuroendocrine neoplasms refractory to prior ^177^Lu therapy, multiple small prospective and real-world series of ^225^Ac-labeled SSTR ligands have reported objective biochemical responses and radiographic disease control together with a generally manageable toxicity profile; the dominant adverse events include xerostomia (from salivary uptake), mild–moderate hematologic suppression, and transient gastrointestinal effects, typically mitigated by activity de-escalation or cycle spacing [23].

**Figure 6:**
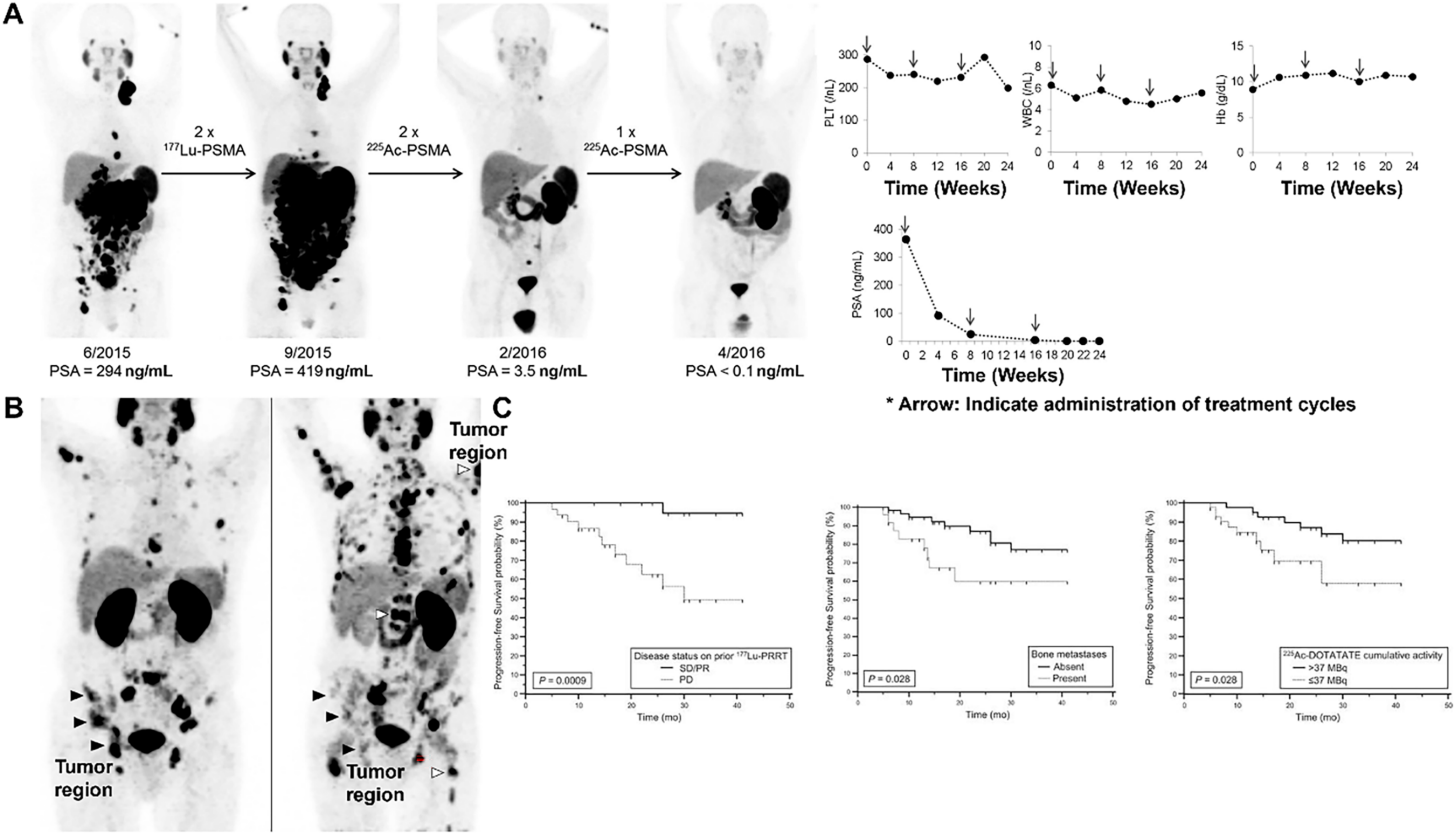
Clinical PSMA-Targeted Radioligand Therapy and Imaging-Based Response Assessment in Metastatic Prostate Cancer. (**A**) ^177^Lu & ^225^Ac-PSMA-617 for PSMA-Targeted α-Radiation Therapy of Metastatic Castration-Resistant Prostate Cancer [6], *Arrow: Indicate administration of treatment cycles. (**B**) Maximum intensity projections of ^68^Ga-PSMA-11 PET in the same patient, broadly representative of the median observed response in the study. The left panel is at baseline and right panel is post 6 cycles of radium-223. Black arrowheads denote example bone metastases showing a partial response on PSMA PET, while the white arrowheads denote some of the newly developed bone metastases [32]. (**C**) Radiologic PFS according to disease status on prior ^177^Lu-PRRT (**Left**), presence or absence of bone metastases (**Middle**), and cumulative activity of ^225^Ac-DOTATATE received (**Right**) [33]. The schematic and figure have been adapted with permission from previously published figures and concepts in references [6,32,33]; copyright 2016, 2022–2023, respective copyright holders. Adapted from Perry E, Talwar A, Sharma S, et al. Non-prostate cancer tumours: incidence on ^18^F-DCFPyL PSMA PET/CT and uptake characteristics in 1445 patients. Eur J Nucl Med Mol Imaging. 2022;49:3277–3288. 10.1007/s00259-022-05721-z. Licensed under the Creative Commons Attribution 4.0 International License (CC BY 4.0) (http://creativecommons.org/licenses/by/4.0/). Reproduced/Adapted with permission from Kratochwil C, et al. J Nucl Med. 2016;57(12):1941–1944. 10.2967/jnumed.116.178673. Reproduced/Adapted with permission from Ballal S, et al. J Nucl Med. 2023;64(2):211–218. 10.2967/jnumed.122.264043

In metastatic castration-resistant prostate cancer, ^225^Ac-PSMA cohorts consistently demonstrate high PSA response rates (with PSA-50 reductions common in responding patients), rapid symptom relief in subsets with visceral disease, and evidence of activity after ^177^Lu exposure, albeit with dose-limiting xerostomia in a fraction of cases; activity selection and salivary-sparing strategies remain active areas of protocol refinement [6,23,32] (Fig. 6A–C). Overall, Fig. 6 integrates representative imaging and outcome analysis to show how targeted radionuclide therapies can be evaluated by molecular response on PET and by progression-free survival stratified by prior treatment history and metastatic burden.

^212^Pb programs extend α-therapy into peptide and small-molecule vectors that benefit from generator availability (e.g., ^224^Ra/^212^Pb generators) and the favorable half-life/chemistry balance of Pb^2+^ for DOTA-type chelation. Early studies with ^212^Pb-labeled SSTR and PSMA constructs report promising anti-tumor signals with short-lived marrow effects as the main systemic toxicity, reflecting the nuclide’s rapid decay chain; optimized infusion logistics and organ dosimetry are central to ongoing development [23,33].

Japan has led first-in-human ^211^At initiatives. The investigator-initiated Alpha-T1 trial of sodium astatide (^211^At-NaAt) in radioiodine-refractory differentiated thyroid cancer completed with acceptable tolerability and preliminary efficacy signals, supported by predictable thyroidal/basal uptake and dosimetric feasibility [19,21,23].

In parallel, [^211^At] PSMA-5 programs have launched to test PSMA-directed α-therapy within controlled dose-escalation frameworks, leveraging astatination chemistry that maintains immunoreactivity while delivering short high-LET tracks suitable for micrometastatic disease [20,23].

Across isotopes, industrialization of α-emitter supply chains—including scaling of ^225^Ac (historically limited by ^229^Th stock) and domestic cyclotron production of ^211^At via the ^209^Bi(α,2n) ^211^At route is expected to alleviate long-standing bottlenecks and enable multi-center, adequately powered studies [19,23,33] (Fig. 6C).

Prospective registries and coordinated trial portfolios now catalog α-therapy across solid and hematologic malignancies, illustrating a broad target spectrum—PSMA, GRPR, HER2, CEA, CD33, among others—and providing templates for protocol design (dose-escalation schemas, salivary/renal protection strategies, response/PRO endpoints) and safety monitoring (xerostomia grading, marrow reserve thresholds, organ-specific dose caps). These resources are accelerating convergence on reporting standards and eligibility criteria, facilitating comparisons across vectors and isotopes and informing the next generation of randomized studies [23].

### 4.3 Vector Classes and Design Trade-Offs

#### 4.3.1 Pharmacologic Scale and Tissue Kinetics

Vector size largely dictates how a radiopharmaceutical distributes, clears, and deposits dose in tumors vs. normal organs. Small molecules (≤1 kDa) diffuse rapidly and penetrate heterogenous tumor parenchyma efficiently, but their fast renal clearance and transporter-mediated uptake can amplify kidney and salivary exposure when targets or off-target transporters are present; short blood residence enables quicker imaging and repeat dosing but may limit dwell time in poorly perfused regions [12,23]. Peptides (1–5 kDa) typically balance penetration with sufficient residence to support high target-to-background ratios; antagonist designs (e.g., GRPR, SSTR) favor binding-site saturation across a larger receptor pool (internalized and non-internalized conformers), often improving tumor-to-organ ratios compared with agonists that rely on receptor internalization alone [12,23]. Antibodies (~150 kDa) and fragments (F(ab^′^)_2_, Fab, scFv, minibodies) provide high specificity and prolonged exposure—useful for targets with slow internalization—but at the cost of slower blood clearance, potential marrow dose accrual, and delayed optimal imaging time points; engineering smaller fragments or Fc-modified scaffolds can accelerate kinetics while preserving affinity [12,23].

#### 4.3.2 Internalization Biology and Microdosimetry

Whether a vector internalizes (and to which compartment) shapes how β, α, or Auger emissions couple to DNA damage. Internalizing ligands can escort nuclides closer to nuclear DNA, enhancing the effect of short-range electrons (e.g., from ^161^Tb) or Auger cascades; non-internalizing antagonists may still outperform *in vivo* if they access a larger receptor pool and improve areal coverage of heterogeneous lesions, leveraging β-particle cross-fire when subcellular proximity is less critical [12,23]. Consequently, programs now match emission type of trafficking profile (e.g., α/Auger with strongly internalizing vectors; β with high-occupancy antagonists) to optimize tumor control while minimizing normal-organ dose [12,23].

#### 4.3.3 Chelation and Radiochemistry Constraints

Choice of chelator/linker governs labeling yield, *in vivo* stability, and off-target retention. For ^177^Lu/^161^Tb, DOTA remains the workhorse given robust thermodynamic stability and established, GMP-compatible protocols across small molecules and peptides; identical DOTA chemistry facilitates isotope switching (Lu ↔ Tb) for side-by-side evaluation of microdosimetric advantages without re-engineering the vector [12,23]. For lead-based platforms (e.g., ^212^Pb as an *in vivo* α-generator), chelators optimized for Pb^2+^ coordination are used to limit transchelation and daughter migration; linker hydrophilicity and charge are tuned to attenuate hepatobiliary retention and improve renal handling [23]. Antibody formats often require residualizing labels and catabolite-aware linkers to minimize non-target organ trapping following lysosomal degradation [12,23].

#### 4.3.4 Organ-at-Risk (OAR) Liabilities and Mitigation

Vector class influences which OARs dominate the therapeutic index. Small molecules targeting PSMA-like antigens may load salivary glands and kidneys; peptides directed to GRPR/SSTR can show pancreas/spleen or renal uptake; antibodies risk marrow/hepatic background due to prolonged circulation [12,23]. Design levers include: (i) antagonist switches to boost tumor binding without excessive internalization in OARs; (ii) hydrophilicity/charge adjustments to accelerate non-target washout; (iii) co-infusions or amino-acid–based renal protection (where applicable) and fractionation to respect OAR dose caps; and (iv) activity personalization via dosimetry to preserve efficacy while reducing cumulative OAR burden [12,23].

#### 4.3.5 Translation Heuristics and Early Human Signals

**Figure 7:**
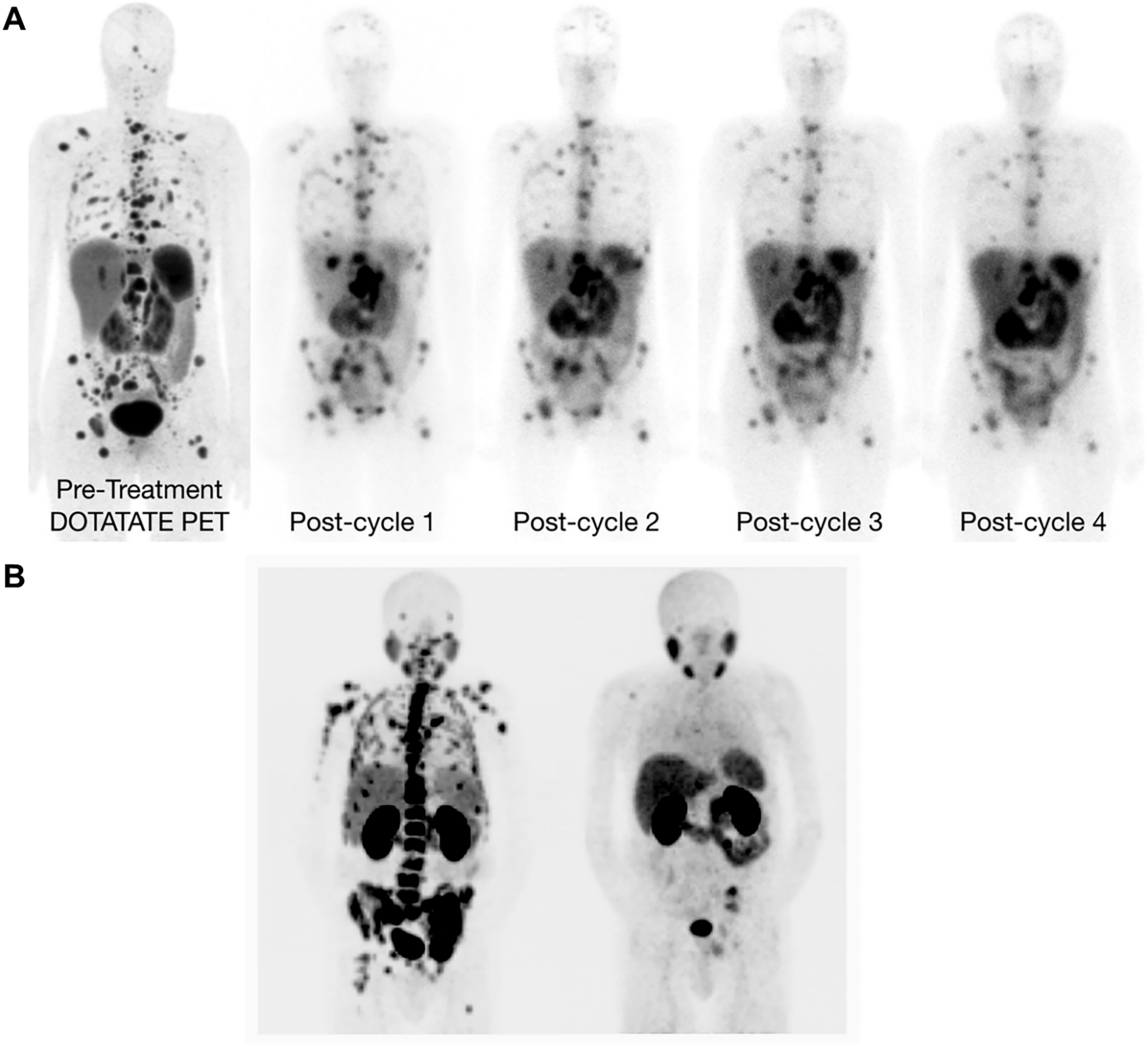
Treatment Monitoring by Whole-Body Imaging in ^177^Lu-Based Radioligand Therapy. (A) Whole-body images acquired after administration of ^177^Lu-DOTATATE in different therapy cycles [12], (**B**) Excellent treatment response after 6 cycles of ^177^Lu-PSMA-617 [34]. The schematic and figure have been adapted with permission from previously published figures and concepts in references [12,34]; copyright 2019–2020, respective copyright holders. Reproduced from Burkett BJ, Bartlett DJ, McGarrah PW, et al. A review of theranostics: perspectives on emerging approaches and clinical advancements. Radiol Imaging Cancer. 2023;5(4):e220157. 10.1148/rycan.220157) © RSNA, 2023 (Open access; journal policy indicates CC BY-NC-ND 4.0.). Reproduced from Pang Y, Zhao L, Meng T, et al. PET imaging of fibroblast activation protein in various tumors using ^68^Ga-FAP-2286: comparison with ^18^F-FDG and ^68^Ga-FAPI-46 in a single-center, prospective study. J Nucl Med. 2023;64(3):386–394. 10.2967/jnumed.122.264544. Licensed under the Creative Commons Attribution 4.0 International License (CC BY 4.0) (http://creativecommons.org/licenses/by/4.0/). © RSNA, 2023 (Open access; journal policy indicates CC BY-NC-ND 4.0.)

Across PSMA, SSTR, and GRPR programs, early patient studies indicate that antagonist peptides can increase tumor uptake and coverage with favorable tumor-to-organ ratios relative to historical agonists, and that DOTA-based small molecules retain the practical advantages (manufacturability, logistics) that enabled widespread adoption of ^177^Lu platforms—now extended to ^161^Tb without major synthetic changes [12,23,34]. Antibody and fragment approaches are advancing where targets exhibit lower density or slower turnover, using longer-lived labels for imaging and carefully titrated therapeutic activities with marrow-sparing schedules [12,23,34]. Collectively, these choices—vector size, internalization profile, chelation chemistry, and OAR-aware engineering—constitute the key design trade-offs that determine whether a promising preclinical signal matures into a clinically sustainable theranostic platform [12,23,34] (Fig. 7). Overall, this figure demonstrates that serial whole-body imaging can capture robust, cycle-dependent therapeutic responses to ^177^Lu-based radioligand therapies across different molecular targets.

## 5 Dosimetry, Quantification, and Response Assessment

### 5.1 Rationale and Current Practice

Although fixed-activity schemas (e.g., four cycles of ^177^Lu-DOTATATE or protocolized activities of ^177^Lu-PSMA-617) remain pragmatic standards in many centers, image-based dosimetry enables patient-specific estimation of organ and lesion absorbed doses and can inform cycle-to-cycle adaptations in responders or in patients approaching organ-at-risk (OAR) limits [9–11]. Contemporary guidance from professional societies encourages harmonized quantitative methods and reporting so that dose estimates are comparable across scanners and sites, and to support trials designed to test individualized activity prescription vs. fixed dosing [9–11,14].

### 5.2 Acquisition and Time–Activity Curves

Multi-time-point (MTP) protocols typically combine planar whole-body surveys (for global kinetics) with quantitative SPECT/CT at selected time points to capture bi-exponential or mono-exponential lesion and organ clearance. Activity is converted to time–activity curves by drawing VOIs on SPECT/CT (or ROIs on planar images with appropriate overlap/attenuation corrections), followed by curve fitting and numerical integration to obtain cumulated activity (Ã) [14]. Dosimetry then proceeds using voxel-based (dose-kernel or Monte-Carlo) methods where available, or MIRD schema/organ-level S-values when voxel methods are not feasible [14]. Practical implementation includes system calibration for absolute quantification, partial-volume recovery for small lesions, and standardized reconstruction to reduce inter-site variability [9–11,14].

### 5.3 Single-Time-Point (STP) and Hybrid Strategies

Because MTP imaging can be resource-intensive, several STP scaling or hybrid approaches have been proposed that anchor an individualized time point to population pharmacokinetics or to a patient-specific planar curve, yielding dose estimates that retain acceptable accuracy for clinical decision-making while minimizing patient burden [14,26]. Comparative analyses show where STP performs well (e.g., for organs/lesions with predictable kinetics) and where MTP remains preferable (e.g., mixed-kinetic lesions or when previous cycles altered clearance), informing protocol selection in routine care vs. trials [14,26].

### 5.4 Organs at Risk and Safeguarding Strategies

**Figure 8:**
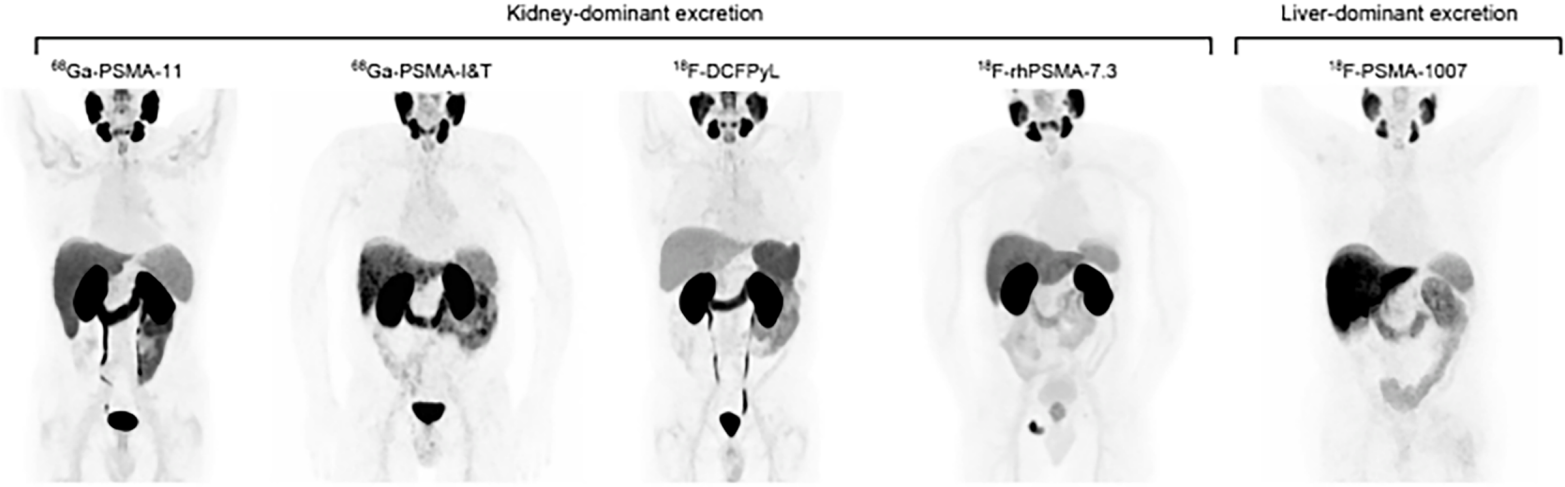
Normal body distribution of PSMA-ligands. [^68^Ga]Ga-PSMA-11, [^68^Ga]Ga-PSMA-I&T, [^18^F]F-DCFPyL, and [^18^F]F-rhPSMA-7.3 applications lead to notable kidney uptake. Bladder retention is high for [^68^Ga]Ga-PSMA-11, [^68^Ga]Ga-PSMA-I&T, and [^18^F]F-DCFPyL and lower for [^18^F]F-rhPSMA-7.3. Reference organs for ligands with kidney-dominant excretion are liver and parotid gland. [^18^F]F-PSMA-1007 leads to high liver uptake due to hepatic excretion. Reference organs for ligands with liver excretion are spleen and parotid gland. Focal uptake in the pelvic bone is noted on the [^18^F]F-rhPSMA-7.3 PET corresponding to metastatic disease [11]. The schematic and figure has been adapted with permission from previously published figures and concepts in reference [11]; copyright 2019. Reproduced from Hope TA, Abbott A, Colucci K, et al. NANETS/SNMMI procedure standard for somatostatin receptor-based peptide receptor radionuclide therapy with ^177^Lu-DOTATATE. J Nucl Med. 2019;60(7):937–943. 10.2967/jnumed.118.230607. Licensed under the Creative Commons Attribution 4.0 International License (CC BY 4.0) (http://creativecommons.org/licenses/by/4.0/)

Quantitative dosimetry focuses on kidneys, salivary glands, liver, spleen, and red marrow as principal OARs, with lesion-level dosimetry used to explore dose–response and biologically effective dose (BED) relationships. Mitigation includes fractionation, cycle spacing, co-infusions (e.g., amino acids for renal protection where appropriate), and activity capping or stopping rules when modeled cumulative doses approach predefined limits [9–11,27]. In practice, many programs employ adaptive continuation (e.g., extended cycles in deep responders) contingent on OAR doses remaining acceptable on interim dosimetry [9–11] (Fig. 8).

### 5.5 Linking Dose to Outcome

For ^177^Lu-PSMA and ^177^Lu-DOTATATE, studies correlating image-derived lesion absorbed dose with biochemical and anatomic/molecular responses suggest that higher per-lesion dose and higher cumulative tumor dose associate with deeper responses and longer control in subsets, though prospective dose-prescription evidence is still emerging [9–11,26]. Ongoing trials are structured to test whether dose-guided personalization improves efficacy–toxicity trade-offs compared with fixed-activity schedules, a central question for broader adoption [9–11,14,26].

### 5.6 Quantitative Response Assessment

Beyond visual criteria, theranostic workflows increasingly incorporate harmonized SUVs (SUV_{peak}/SUV_{mean}), tumor-to-background ratios, and volumetric burden metrics (e.g., PSMA-TV, total lesion PSMA; SSTR-based analogs) as endpoints that can be measured at baseline, early on-treatment, and post-cycle to gauge pharmacodynamic effects [9–11]. On the eligibility and delivery-confirmation ends of the loop, SSTR/PSMA imaging remains integral—first to confirm target expression and suitable biodistribution, and later to verify on-target delivery and interim response, enabling escalation, maintenance, or switch decisions within the same molecular axis [9–11,26].

### 5.7 Operational Essentials for Multi-Center Use

To reduce variability, guidelines emphasize: (i) scanner calibration and EARL-like harmonization for quantitative PET/SPECT; (ii) standardized VOI delineation and reconstruction settings; (iii) reporting templates that include administered activity, imaging time points, reconstruction details, and uncertainty ranges; and (iv) consistent handling of red-marrow dose (blood-based or image-based surrogates) when marrow toxicity is a concern [9–11,26]. These practices underpin reproducible dose reporting and are foundational for trials comparing fixed vs. individualized regimens [9–11,14,26].

## 6 Manufacturing, Supply Chain, and Regulatory Considerations

Scalability, chemistry, and standardization for α-emitters. The supply side remains the dominant rate-limiter for clinical dissemination. For ^211^At, production relies on regional cyclotrons via ^209^Bi(α,2n)^211^At, with yield and reliability governed by target (high-purity bismuth, heat management), beam current, and rapid, GMP-compatible dry-distillation to meet a 7.2-h half-life window; these constraints naturally favor distributed manufacturing hubs close to clinics to preserve specific activity and reduce decay losses [19,23]. For ^225^Ac, historical dependence on ^229^Th/^225^Ac generators has limited batch sizes; ongoing industrialization efforts explore diversified routes and capacity expansion with harmonized release criteria (radionuclide purity, daughter breakthrough, specific activity) to enable multi-center, adequately powered studies [23].

On the radiochemistry front, α-emitters impose distinctive design pressures. Astatination chemistry must maximize *in vivo* stability of the C–At bond and resist deastatination in oxidative/thiol-rich compartments; practical solutions include optimized aryl-tin/boronate precursors and prosthetic groups that preserve immunoreactivity of sensitive vectors while shortening synthesis and purification to fit the ^211^At time budget [19,23]. For ^225^Ac, chelation remains a key bottleneck: macrocyclic platforms must balance complexation kinetics, thermodynamic stability, and daughter-nuclide recoil management to limit off-target retention; process standardization (e.g., buffer systems, temperature, metal contaminants) and batch-release QC (radiochemical purity, endotoxin, sterility) are integral to reproducibility at scale [23].

Logistics and regulation add additional layers: half-life–driven shipping windows (especially for ^211^At), on-site vs. near-site synthesis models, environmental controls for volatile astatine, and validated cleaning/containment protocols are increasingly codified in institutional SOPs and sponsor CMC packages. Regulators also scrutinize daughter in-growth (e.g., ^221^Fr, ^213^Bi from ^225^Ac) and their implications for handling, patient safety, and dosimetry reporting [19,23].

Finally, broad regulatory acceptance hinges on quantitative standardization and robust, patient-specific dosimetry. Harmonized calibration and reconstruction for SPECT/PET used in therapy planning, transparent uncertainty budgets, and convergent reporting frameworks (organ/lesion doses, marrow models, biologically effective dose where appropriate) are repeatedly cited prerequisites for label-enabling trials and cross-site comparability. In this context, evolving EANM–MIRD recommendations and voxel/organ-level workflows provide the methodological substrate for individualized activity prescription and safety monitoring in α-programs as they scale beyond early-phase centers [14,23].

## 7 Expanding Targets and Disease Spaces

### 7.1 Pan-Tumor Expansion beyond PSMA/SSTR

A growing roster of tumor-associated targets is advancing from preclinical validation to early clinical imaging, with several programs showing the attributes needed for radiotheranostic translation. TROP-2 exemplifies this trajectory: first-in-human PET with [^68^Ga] MY6349 demonstrated pan-tumor feasibility with heterogeneous but frequent uptake across multiple histologies, and early on-treatment imaging captured pharmacodynamic response to antibody–drug conjugates (ADCs) in triple-negative breast cancer—supporting roles in patient enrichment, sequence optimization (ADC labeled radioligand), and early response adjudication [23,24]. Nectin-4 tracers (e.g., [^68^Ga] N188) show lesion uptake that correlates with immunohistochemical expression in urothelial carcinoma and detectable signal in other epithelial cancers, positioning Nectin-4 as a drugged target (ADC precedent) that now gains a whole-body imaging readout to map heterogeneity and guide future therapeutic labeling [23].

### 7.2 Pipelines with Theranostic Promise

#### 7.2.1 Fibroblast Activation Protein (FAP/FAPI)

Fibroblast activation protein is highly expressed on cancer-associated fibroblasts in the stroma of many epithelial tumors and selected non-malignant fibroinflammatory diseases, while being largely absent from most normal adult tissues. Quinoline-based FAP inhibitors (FAPI) labeled with ^68^Ga or ^18^F have shown high tumor-to-background ratios across a broad spectrum of cancers, often outperforming ^18^F-FDG for lesion conspicuity and staging in proof-of-concept studies. On the therapeutic side, peptide-based constructs such as FAP-2286 and albumin-binding derivatives (e.g., ^177^Lu-EB-FAPI) exhibit prolonged tumor retention and encouraging early signs of disease control in heavily pretreated patients, positioning FAP/FAPI as a prototypical pan-tumor stromal target for radiotheranostics [FAP1–FAP4]. Key challenges include balancing marrow and renal dose with the need for durable stromal irradiation, managing uptake in benign fibroinflammatory conditions, and defining response criteria when FAP expression reflects both tumor burden and microenvironmental remodeling [35–39].

#### 7.2.2 Gastrin-Releasing Peptide Receptor (GRPR)

GRPR is a bombesin-family G-protein–coupled receptor overexpressed in prostate, breast, lung, and gastrointestinal cancers, with relatively limited expression in most normal adult tissues. Antagonist-based tracers such as ^68^Ga-RM2, ^68^Ga-AMTG and related ligands have demonstrated specific uptake and correlation with hormone-receptor status in estrogen-receptor–positive breast cancer and prostate cancer, supporting their use for lesion detection, staging, and restaging [GRPR1–GRPR3]. Preclinical and early clinical studies of ^177^Lu-labeled GRPR antagonists indicate favorable tumor retention and manageable off-target uptake, particularly when pancreatic and gastrointestinal doses are carefully monitored, highlighting GRPR as a candidate for radiotheranostic applications in hormone-driven and neuroendocrine malignancies [GRPR2, GRPR4]. However, receptor down-regulation with prior endocrine or systemic therapy, as well as physiologic uptake in pancreas and bowel, necessitate thoughtful timing of imaging and therapy and may motivate combination strategies with radiosensitizers [40–44].

#### 7.2.3 HER2-Directed Imaging and Therapy

HER2 is a validated oncogenic driver and therapeutic target in breast, gastric, and other solid tumors. Radiolabeled trastuzumab and pertuzumab antibodies, most commonly with ^89^Zr for PET, have enabled noninvasive mapping of HER2 expression and heterogeneity, including detection of HER2-positive metastases in patients with HER2-negative primaries and prediction of response to antibody–drug conjugates [HER2-1, HER2-2]. Early-phase studies of ^177^Lu-trastuzumab and small-protein scaffolds (e.g., HER2-targeted affibodies) suggest that HER2-targeted radiotheranostics can be delivered safely with lesion-specific uptake, particularly in patients with refractory disease after standard HER2-directed systemic therapies [HER2-2, HER2-3]. Integration into clinical practice will require careful attention to cardiotoxicity risk, competition with existing HER2-directed drugs, and development of trial designs that exploit HER2-PET both for patient selection and on-treatment response assessment [45–47].

#### 7.2.4 LAT1 (SLC7A5)

Overexpressed across many solid tumors and linked to metabolic rewiring, LAT1-directed tracers exploit amino-acid transport biology to achieve high tumor-to-background ratios. If normal-organ kinetics (liver, pancreas, kidneys) remain permissive, LAT1 could support broad, tissue-agnostic radioligand strategies, particularly with β/mixed-electron emitters that leverage cross-fire in heterogeneous lesions [23].

#### 7.2.5 GPC-1

Glypican-1 is a cell-surface heparan sulfate proteoglycan with reported up-regulation in pancreatic and other epithelial malignancies. Early PET probes indicate targetable biology, and therapy translation will hinge on internalization and catabolite handling to maintain therapeutic index in hepatobiliary-clearance settings [23].

#### 7.2.6 EphA2

EphA2 is a receptor tyrosine kinase implicated in invasion, metastatic spread, and therapeutic resistance. Imaging probes are progressing toward clinical feasibility, and paired ^89^Zr/^177^Lu antibody constructs have shown proof-of-concept for fully integrated imaging and therapy. Pairing EphA2 targeting with α-emitters may be attractive for microinvasive fronts and minimal residual disease, provided normal-tissue expression (e.g., endothelium, selected epithelia) remains manageable [23, EphA2-1].

#### 7.2.7 CA IX

Carbonic anhydrase IX is a hypoxia-linked enzyme enriched in clear-cell renal cell carcinoma and hypoxic niches in other tumors. Antibody- and small-molecule–based PET tracers can delineate hypoxic tumor subcompartments in which high-LET or mixed-electron emitters may outperform β-only approaches [CAIX-1, CAIX-2]. Translation to routine therapy will require careful renal dose management and verification that image-positive, hypoxic volumes receive adequate delivered dose, potentially through voxel-based or microdosimetric planning.

#### 7.2.8 From Imaging to Therapy: Decision Points

For each target, translation follows a common pathway: (i) demonstrate robust PET signal with correlation to tissue expression and pathway activity; (ii) characterize normal-organ kinetics to define organs-at-risk (e.g., kidneys, liver, salivary and exocrine glands); (iii) establish repeatability and quantitative cut-points (SUV_peak, tumor-to-blood/background ratios) tied to clinical outcomes; and (iv) perform dosimetry-feasibility studies with the intended therapeutic label (β, α, or mixed-electron) to confirm a workable therapeutic index in image-positive disease [23,24]. Practical pitfalls include on-target uptake in exocrine glands (salivary/pancreas), hepatobiliary retention for lipophilic constructs, and dynamic target modulation under prior therapies; each requires vector engineering (antagonists, linker polarity, albumin binding), schedule adjustments, or organ-protection strategies to sustain cumulative dosing [23].

#### 7.2.9 Trial Architectures That Fit Pan-Tumor Biology

The broader precision-oncology shift toward master protocols and basket designs—which enroll by biomarker rather than anatomic site—offers efficient blueprints to evaluate these targets across diverse histologies. Imaging can serve both as a binary enrichment tool (positivity threshold) and as a quantitative endpoint (baseline and early on-treatment change), while small, histology-specific expansion cohorts preserve signal detection and safety characterization. Such architectures accelerate generalizability, enable head-to-head comparisons of vector classes or isotopes within a target, and permit adaptive randomization once early efficacy signals emerge [1].

#### 7.2.10 Outlook

Collectively, FAP/FAPI, GRPR, HER2, TROP-2, Nectin-4, LAT1, GPC-1, EphA2, and CA IX illustrate a shift toward broadly expressed oncogenic and microenvironmental programs that are compatible with tissue-agnostic radiotheranostics. As target-positive populations are defined by whole-body PET and dosimetry confirms deliverable dose to image-positive lesions with acceptable organ exposure, these axes can extend radiopharmaceutical therapy beyond legacy PSMA/SSTR indications, aligning clinical research with the biomarker-first paradigm that now anchors modern drug development [1,23,24].

## 8 Challenges and Future Directions

Key challenges cluster around three domains. First, biology and targeting: intra- and inter-lesional heterogeneity, target-negative disease compartments, and organ-specific off-target uptake (e.g., kidneys, salivary glands, pancreas with GRPR agents) complicate durable control and mandate careful vector engineering and toxicity mitigation [12,23].

Second, quantification and personalization: routine, standardized dosimetry remains uncommon but is likely necessary to optimize therapeutic indices, especially as repeat/extended cycles and combination regimens proliferate. Prospective trials explicitly comparing fixed vs. individualized activity are critical to move from plausibility to practice [9–11,26,28].

Third, infrastructure and access: reliable isotope supply chains, reproducible radiochemistry, and harmonized quantitative imaging are essential to scale α-therapy and next-generation β/mixed-electron platforms beyond select centers. Continued industrialization, guideline maturation, and multi-disciplinary care pathways will determine the speed and equity of adoption [19,23].

## 9 Conclusions

Radiotheranostics has transitioned from a pair of successful axes (SSTR and PSMA) into a broader, modality-agnostic framework that links whole-body target confirmation with image-guided therapy, dose optimization, and longitudinal response assessment. On the physics side, clear divisions in LET and range among β, α, and Auger emissions now inform indication selection and vector design: β-emitters remain practical for heterogeneous, millimeter-scale disease via crossfire, while α-emitters and mixed-electron nuclides promise superior microdosimetry for micrometastases, hypoxic niches, and small-volume lesions. Within this landscape, ^177^Lu continues to anchor clinical practice, but ^161^Tb has emerged as a plug-compatible alternative whose additional low-energy electron component may improve cellular-scale dose without sacrificing operational simplicity.

Diagnostics are keeping pace. Non-FDG PET programs—exemplified by TROP-2 and Nectin-4—demonstrate pan-tumor feasibility, early pharmacodynamic readouts, and a clear runway to theranostic translation when biodistribution and organ risk are favorable. Parallel pipelines in FAP/FAPI, GRPR, HER2, LAT1, GPC-1, EphA2, and CA IX further support a shift toward tissue-agnostic targeting, with PET providing the quantitative gatekeeper for enrichment, sequencing, and real-time adaptation. As these targets mature, success will hinge on aligning emission type with trafficking biology (e.g., α/Auger for strongly internalizing vectors; β for high-occupancy antagonists) and on deploying linker/chelator engineering to stabilize labels and reduce off-target retention.

Personalization through dosimetry is the next inflection point. Multi-time-point quantitative imaging with planar/SPECT/CT and voxel- or organ-level methods can individualize activity, protect organs at risk, and clarify dose–response relationships—yet remains unevenly adopted. Practical single-time-point and hybrid workflows, combined with harmonized calibration and reconstruction, offer a credible path to routine use. Prospective trials that compare fixed-activity vs. dose-guided regimens will determine whether individualized prescriptions translate into better efficacy–toxicity trade-offs at scale.

Manufacturing and regulation are equally determinative. Industrialization of ^225^Ac supply and regional cyclotron production of ^211^At are beginning to ease long-standing bottlenecks, but consistent radionuclide purity, robust chelation (including daughter recoil management), and GMP-compatible, time-efficient chemistry—especially for astatine—remain prerequisites for widespread access. Regulators increasingly expect transparent uncertainty budgets, standardized reporting of organ/lesion doses, and integration of patient-specific dosimetry into study design, bringing internal radiation therapy closer to external-beam radiotherapy’s quantitative rigor.

Clinically, targeted α-therapy has crossed a credibility threshold with activity signals in ^225^Ac-PSMA cohorts and early ^211^At programs, while ^212^Pb platforms extend the toolkit with generator-enabled logistics. Toxicities such as salivary and renal exposure, marrow dose, and pancreas uptake in GRPR pathways will require continued vector engineering, organ protection strategies, and adaptive scheduling to preserve the therapeutic index over repeated cycles and combinations.

Looking forward, radiopharmaceuticals are poised to become a true decision platform in oncology: the same molecular axis that stages disease will guide therapy selection, personalize activity, and adjudicate response. To realize that vision, the field’s priorities are clear: (i) complete the supply-chain build-out for α-emitters while standardizing release/QC criteria; (ii) normalize quantitative dosimetry and uncertainty reporting across centers; (iii) advance vector engineering that reconciles tumor coverage with organ protection; (iv) embed PET-based enrichment and early pharmacodynamic endpoints into basket/master-protocol architectures; and (v) rigorously test combinations (e.g., with DNA-damage response agents or immunotherapy) under dose-aware designs. If these strands converge, next-generation radiotheranostics—spanning ^161^Tb, ^211^At, ^225^Ac, and beyond—can extend well past legacy indications and help define a biomarker-first standard of care across diverse cancers.

## Data Availability

The datasets used and/or analyzed during the current study are available from the corresponding authors on reasonable request.
